# Baclofen Protects Primary Rat Retinal Ganglion Cells from Chemical Hypoxia-Induced Apoptosis Through the Akt and PERK Pathways

**DOI:** 10.3389/fncel.2016.00255

**Published:** 2016-11-04

**Authors:** Pingping Fu, Qiang Wu, Jianyan Hu, Tingting Li, Fengjuan Gao

**Affiliations:** ^1^Department of Ophthalmology, Shanghai Jiao Tong University Affiliated Sixth People’s HospitalShanghai, China; ^2^Shanghai Key Laboratory of Diabetes Mellitus, Shanghai Jiao Tong University Affiliated Sixth People’s HospitalShanghai, China

**Keywords:** apoptosis, retinal hypoxia disease, retinal ganglion cells, baclofen, GABA_B_ receptor, cobalt, ER stress

## Abstract

Retinal ganglion cells (RGCs) consume large quantities of energy to convert light information into a neuronal signal, which makes them highly susceptible to hypoxic injury. This study aimed to investigate the potential protection by baclofen, a GABA_B_ receptor agonist of RGCs against hypoxia-induced apoptosis. Cobalt chloride (CoCl_2_) was applied to mimic hypoxia. Primary rat RGCs were subjected to CoCl_2_ with or without baclofen treatment, and RNA interference techniques were used to knock down the GABA_B_2 gene in the primary RGCs. The viability and apoptosis of RGCs were assessed using cell viability and terminal deoxynucleotidyl transferase-mediated dUTP nick end-labeling (TUNEL) assays, Hoechst staining, and flow cytometry. The expression of cleaved caspase-3, bcl-2, bax, Akt, phospho-Akt, protein kinase RNA (PKR)-like ER kinase (PERK), phospho-PERK, eIF2α, phospho-eIF2α, ATF-4 and CCAAT/enhancer-binding protein homologous protein (CHOP) were measured using western blotting. GABA_B_2 mRNA expression was determined using quantitative real-time polymerase chain reaction (qRT-PCR) analysis. Our study revealed that CoCl_2_ significantly induced RGC apoptosis and that baclofen reversed these effects. CoCl_2_-induced reduction of Akt activity was also reversed by baclofen. Baclofen prevented the activation of the PERK pathway and the increase in CHOP expression induced by CoCl_2_. Knockdown of GABA_B_2 and the inactivation of the Akt pathway by inhibitors reduced the protective effect of baclofen on CoCl_2_-treated RGCs. Taken together, these results demonstrate that baclofen protects RGCs from CoCl_2_-induced apoptosis by increasing Akt activity and by suppressing the PERK pathway and CHOP activation.

## Introduction

The retina is the most metabolically active tissue in human body (Ames, 1992; Caprara and Grimm, 2012). In retinal disease such as retinopathy of prematurity, diabetic retinopathy, age-related macular degeneration and glaucoma, an insufficient supply of oxygen or nutrients, may occur during conditions of disturbed hemodynamics or vascular defects, results in highly impaired cellular oxygen balance, and retinal neurons become hypoxic (Kaur et al., 2009, 2012; Munemasa and Kitaoka, 2012). Among the retinal neurons, retinal ganglion cells (RGCs) are the predominant components that undergo hypoxic injury (Abu-El-Asrar et al., 2004; Caprara and Grimm, 2012; Munemasa and Kitaoka, 2012), for they are the ultimate neurons in retina that consume large quantities of energy to convey visual information to the brain (Sanes and Masland, 2015). Hypoxia-related disorders enhance or induce the death of RGCs in the retina, resulting in irreversible vision loss, most of which through apoptosis (Kaur et al., 2009; Yang et al., 2013).

Several hypoxic chemicals have been used to explore the molecular events that underlie the hypoxic neuronal death (Kim et al., 2015). Among all the divalent cations that act as hypoxic mimetics, Cobalt chloride (CoCl_2_) has shown to induce hypoxia (Yan et al., 2005; Cascio et al., 2008; Law et al., 2012; Lopez-Sánchez et al., 2014) by increasing HIF1α stability and degrading HIF1α (Carbajo-Pescador et al., 2013; Kim et al., 2015; Masoud and Li, 2015). In the present study, RGCs were treated with CoCl_2_ to stimulate hypoxia.

Gamma-aminobutyric acid (GABA) is the main inhibitory neurotransmitter involved in neuronal information processing within the retina (Koulen et al., 1998), and ganglion cells, along with other types of retinal neurons receiving input from GABAergic cells (Nag and Wadhwa, 1997; Koulen et al., 1998). GABA mediates its actions via distinct receptor systems, including the ionotropic GABA_A_ and GABA_C_ receptors and the metabotropic GABA_B_ receptor. The GABA_B_ receptor belongs to the class C G-protein-coupled receptors and is composed of two subunits—GABA_B_1 and GABA_B_2—both of which are required for normal receptor function (Bettler et al., 2004; Liu et al., 2004; Pin et al., 2004).

Recent advances indicate that GABA can act beyond its classical role in synaptic communication and may modulate nearly all critical steps of neuronal network formation including neurodegenerative, neuroinflammatory and neuromigration (Gaiarsa and Porcher, 2013; Crowley et al., 2015). Accumulating evidences have shown that specific activation of GABA_B_ receptor can protect neurons from apoptosis under metabolic stress (Tu et al., 2010; Naseer et al., 2013; Shilpa et al., 2013). Baclofen is a stereoselective GABA_B_ receptor agonist that reduces the release of excitatory neurotransmitters and substances (Albright, 1996; Krach, 2001). It is a drug approved by the Food and Drug Administration that has been used to control spasticity (Furr-Stimming et al., 2014; Crowley et al., 2015) and in the treatment of pain management (Ward and Kadies, 2002) and alcohol addiction (Howland, 2012). Studies have demonstrated a neuroprotective effect of baclofen through the activation of GABA_B_ receptor (Babcock et al., 2002; Tyurenkov et al., 2012; Naseer et al., 2013). It has been reported that the upregulation of GABA_B_ receptor activity could inhibit NMDA-receptor-mediated nitric oxide (NO) production by neuronal NO synthase (nNOS) in brain ischemic injury (Zhou et al., 2008). Recent studies have revealed that, under chronic cerebral hypoperfusion, the effects of GABA_B_ receptors activated by baclofen could reverse neuronal damage by increasing the activation of Akt, GSK-3β and ERK and upregulating the bcl-2/bax ratio, which suppress cytodestructive autophagy (Liu et al., 2015). Another study has shown that baclofen markedly improves memory impairment and alleviates neuronal damage in chronic ischemic rats by increasing the expression and regulating the function of the TPR-containing Rab8b-interacting protein (TRIP8b; Li et al., 2014b).

Given previous reports linking protective effects of baclofen and neurons with hypoxic injuries, particularly with relevance to neuron apoptosis, it is therefore rational to hypothesized that baclofen might have similar neuroprotective effects on RGCs undergo hypoxic stress. In the present investigation, we explored the impact of baclofen on the cobalt-induced hypoxic RGCs, using primary rat ganglion cells isolated from neonatal Sprague-Dawley (SD) rats. In addition, the relevant molecular pathways associated with the neuroprotective effects of baclofen were evaluated.

## Materials and Methods

### Purified Rat Retinal Ganglion Cell Culture

All animal experiments were approved by the Animal Experimentation Ethics Committee of the Sixth People’s Hospital, Shanghai Jiao Tong University and were carried out in accordance with the approved guidelines for the Care and Use of Laboratory Animals of the National Institutes of Health (Bethesda, MD, USA). Primary RGC cultures were established as previously described (Yamagishi and Aihara, 2014), with some modification. Briefly, retinas were dissected from the eyes of 1- to 4-day-old SD rats (SIPPR/BK Lab Animal Ltd, Shanghai, China). This was followed by a two-step immunopanning procedure, as described below (Yamagishi and Aihara, 2014). Retinal tissues were incubated at 37°C for 30 min in a solution containing 5 mg/ml papain (all reagents were obtained from Sigma Aldrich, St Louis, MO, USA, unless otherwise stated) in minimum essential medium (MEM; Gibco, Grand Island, NY, USA). To obtain a single cell suspension, tissues were then sequentially triturated through a narrow-bore Pasteur pipette in an ovomucoid solution. After centrifugation at 400× g for 10 min, cells were resuspended in 0.1% bovine serum albumin (BSA) in MEM. Antibodies were then removed, and the cell suspension was incubated in an anti-macrophage antibody-coated (Abcam, Cambridge, MA, USA) flask for 1 h at room temperature and in an anti-Thy1.1 antibody-coated (Abcam, Cambridge, MA, USA) flask for 1 h at 37°C. Cells adhering to the flask (RGCs) were resuspended in a base medium (Neurobasal medium (Gibco, Grand Island, NY, USA) containing 2% B27, 0.1 mg/ml BSA, 0.1 mg/ml transferrin, 1 mM L-glutamine, 5 μg/ml insulin, 1 mM sodium pyruvate, 40 ng/ml triiodothyronine, 40 ng/mL thyroxine, 60 ng/ml progesterone, 16 μg/ml putrescine, 40 ng/ml sodium selenite, 60 μg/ml N-acetyl cysteine, 50 ng/ml brain derived neurotrophic factor (BDNF), 10 ng/ml basic fibroblast growth factor (bFGF), 10 ng/ml ciliary-derived neurotrophic factor (CNTF), 5 mM Forskolin, 100 units/mL penicillin, and 100 mg/mL streptomycin), seeded onto plates that had been coated with 0.05 mg/ml poly-L-lysine overnight, and rinsed twice with sterile deionized water. RGCs were cultured for 48 h at 37°C in a humidified incubator with 5% CO_2_ in base medium before each experiment. Cultures were washed once with phosphate-buffered saline (PBS; Gibco, Grand Island, NY, USA) and pre-incubated at 37°C in neurobasal medium for 60 min before drug treatment. Drugs were freshly prepared and dissolved in serum-free neurobasal medium containing 2% B27. Drug treatments were as follows: MK-2206 2HCl (5 μM, 24 h; Selleckchem, Houston, TX, USA); CoCl_2_ (for the indicated concentrations and times); baclofen (for the indicated concentrations and times).

### Cell Viability Assay

Cell viability was measured using Cell Counting Kit-8 (CCK-8; Dojindo, Japan) according to the manufacturer’s instructions. The CCK-8 assay was performed in a 96-well culture plate with three replicates for each condition at an absorbance of 450 nm. The number of living cells in each well was expressed as the value relative to the control.

### Small Interfering RNA Transfection

Small interfering RNA (siRNA) targeted against GABA_B_2 and scrambled small hairpin RNA (shRNA) were designed and packaged by Genechem Co., Ltd (Shanghai, China). The sequences used were as follows: rat GABA_B_2 siRNA1 (5′-CCA AGG ACA AGA CCA UCA UTT-3′), siRNA2 (5′-CCA AAC AAA UCA AGA CCA UTT -3′), siRNA3 (5′-CCG AGU GUG ACA AUG CAA ATT-3′), and negative control (5′-UUC UCC GAA CGU GUC ACG UTT -3′). Transfection was performed with Lipofectamine 2000 (Thermo Fisher Scientific, Shanghai, China) according to the manufacturer’s instruction. Forty-eight hours after transfection, the medium was replaced and the cells were treated with CoCl_2_ and baclofen as described above.

### TUNEL Assay

Terminal deoxynucleotidyl transferase-mediated dUTP nick end-labeling (TUNEL) staining was used to detect apoptosis-specific nuclear DNA fragmentation. Cells were fixed in 4% paraformaldehyde for 20 min at room temperature before TUNEL staining. The fixed cells were then stained using a commercial TUNEL kit (*In Situ* Cell Death Detection Kit; Boster, Wuhan, China) according to the manufacturer’s recommended instructions. TUNEL-positive cell nuclei were visualized based on green fluorescence and imaging was performed under 40× magnification. The percentage of TUNEL-positive cells was calculated in five microscopic fields from each slide. The population of TUNEL-positive cells was also quantified by flow cytometry.

### Hoechst Stain

After being fixed with 4% paraformaldehyde at 4°C for 30 min, cells were incubated with the DNA stain Hoechst 33,342 (Beyotime, Shanghai, China) for 10 min at room temperature and mounted with a fluorescent mounting medium (Beyotime, Shanghai, China). Images were acquired using a fluorescence microscope (Olympus, Shinjuku, Japan).

### Apoptosis Detection by Flow Cytometry

Apoptosis was measured using a fluorescein isothiocyanate (FITC) Annexin V Apoptosis Detection Kit (BD Biosciences, San Diego, CA, USA) according to the manufacturer’s instructions. Cells were incubated in trypsin-EDTA, collected and suspended in 1× annexin V binding buffer. One hundred microliters of each cell suspension was incubated with 5 μl annexin V-FITC and 5 μl propidium iodide (PI) at room temperature in the dark for 15 min. Following this incubation, 400 μl of 1× binding buffer was added to each tube, and samples were analyzed using FACS. Each experiment was repeated in triplicate.

### Western Blot Analysis

After each treatment, RGCs were harvested and lysed in RIPA buffer (1% Nonidet P-40, 0.5% sodium deoxycholate, 0.1% SDS in PBS) and centrifuged at 12,000 rpm for 20 min at 4°C. Protein was extracted, and equal amounts of protein (30 μg per lane) were separated by 8% or 10% SDS-PAGE and transferred onto polyvinylidene fluoride membranes (Millipore). The membranes were blocked with 5% nonfat milk for 1 h and then incubated overnight at 4°C with the following primary antibodies (all antibodies were obtained from Cell Signaling Technology Inc., Danvers, MA, USA, unless otherwise stated): rabbit anti-cleaved caspase-3 (1:1000), rabbit anti-bcl-2 (1:1000), rabbit anti-bax (1:1000), rabbit anti-Akt (1:1000), mouse anti-phospho-Akt (1:1000), rabbit anti-protein kinase RNA (PKR)-like ER kinase (PERK) (1:1000), rabbit anti-phospho-PERK (1:1000), rabbit anti-eIF2α (1:1000), rabbit anti-phospho-eIF2α (1:1000), rabbit anti-ATF-4 (1:1000), and rabbit anti-CHOP (1:1000; Abcam, Cambridge, MA, USA). After the membranes were washed, a goat anti-rabbit or goat anti-mouse horseradish peroxidase-conjugated secondary antibody (1:2000; Proteintech Group, Chicago, IL, USA) was applied, and proteins were visualized using an enhanced chemiluminescence detection system (Bio-Rad, Hercules, CA, USA). Band intensity was analyzed with Quantity One version 4.6.2 software (Bio-Rad) and compared with the α-tubulin (1:200; Boster, Wuhan, China) internal standard.

### RT-PCR

Harvested cells were washed with PBS, and total RNA was extracted from cells using Trizol reagent (Invitrogen, Carlsbad, CA, USA). The primer sequences used were as follows: rat GABA_B_2 (forward: 5′-CGG AGG ACA GTG GAG AGG TA-3′, reverse: 5′-AGA CAA TGC CAA GCC AGA TG-3′), and rat β-actin (forward: 5′-CAC CCG CGA GTA CAA CCT TC-3′, reverse: 5′-CCC ATA CCC ACC ATC ACA CC-3′). Quantitative real-time polymerase chain reaction (qRT-PCR) was performed using the SYBR Green quantitative PCR (qPCR) Super Mixture (Takara, Tokyo, Japan) and the ABI Prism 7500 Sequence Detection System (Applied Biosystems, Foster City, CA, USA). All reactions were performed in triplicate. The data were analyzed using the 2^−ΔΔCT^ method.

### Statistical Analysis

All experiments were repeated at least three times, and representative results are shown. The differences between two groups were compared with an independent-sample *t*-test (SPSS 19.0 software, Chicago, IL, USA). A two-tailed *P* < 0.05 was considered to indicate statistical significance. Data are presented as the means ± standard deviation (SD).

## Results

### CoCl_2_ Induces RGC Apoptosis

**Figure 1:**
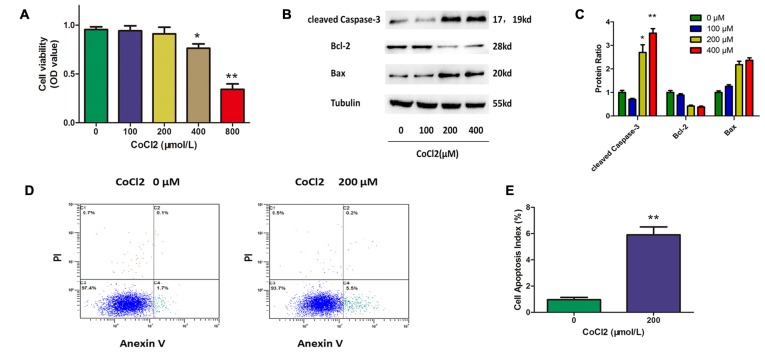
**Cobalt chloride (CoCl_2_) induces retinal ganglion cells (RGCs) apoptosis. (A)** Cell viability detected by Cell Counting Kit-8 (CCK-8) assay in RGCs treated with indicated concentrations of CoCl_2_ for 24 h. Data are means ± SD of three independent experiments. *p < 0.05, **p < 0.01 vs. basal level. **(B,C)** Expression of cleaved caspase-3, bax and bcl-2 detected by Western blotting in RGCs treated with indicated concentrations of CoCl_2_ for 24 h. The corresponding densitometric analyses of the protein bands detected in the immunoblots and normalized to the signal of α-tubulin are also shown. The level of protein in each group was expressed as the value relative to the control. Data are means ± SD of three independent experiments. **p < 0.01, compared with the control. **(D,E)** Cell apoptosis detected by Annexin V and propidium iodide (PI) staining methods in RGCs treated with indicated concentrations of CoCl_2_ for 24 h. The C3 quadrant (Annexin V−/PI−), C4 quadrant (Annexin V + /PI−) and C2 quadrant (Annexin V + /PI+) indicate the percentage of viable cells, apoptotic cells and necrotic cells, respectively. The percentage of apoptotic cells following CoCl_2_ treatment compared with the control group. Values represent the mean ± SD of three independent experiments. **P < 0.01.

To establish a cell model for this study, we first assessed the apoptotic response of RGCs to hypoxia-mimetic agent cobalt. Different concentrations of CoCl_2_ (0, 100, 200, 400, 800 μM) were added to the RGC culture medium. The CCK-8 assay was used to determine cell viability, and the results showed that cell viability was significantly decreased after 24 h of exposure to CoCl_2_ in a dose-dependent manner (Figure 1A). Western blot analysis indicated that CoCl_2_ stimulation not only increased the level of cleaved caspase-3 and bax proteins, but also decreased the level of bcl-2 protein in a dose-dependent manner (Figures 1B,C). We noticed that, at a concentration of 200 μM of CoCl_2_, RGC viability was not significantly impaired even though the level of apoptosis-related proteins had been obviously changed. Furthermore, annexin V/PI double-stained flow cytometry was used to evaluate the CoCl_2_-induced apoptosis in RGCs. Annexin V-positive and PI-negative cells were considered to be the apoptotic cells. After treatment with 200 μM CoCl_2_ for 24 h, the percentage of apoptotic cells increased significantly compared with the percentage following treatment with 0 μM CoCl_2_ (*P* < 0.01, Figures 1D,E). Once our experimental conditions were set up and the use of CoCl_2_ as an effective apoptosis inductor confirmed, the 200 μM concentration was chosen for further investigations.

### Baclofen Protected RGCs Against CoCl_2_–Induced Apoptosis

**Figure 2:**
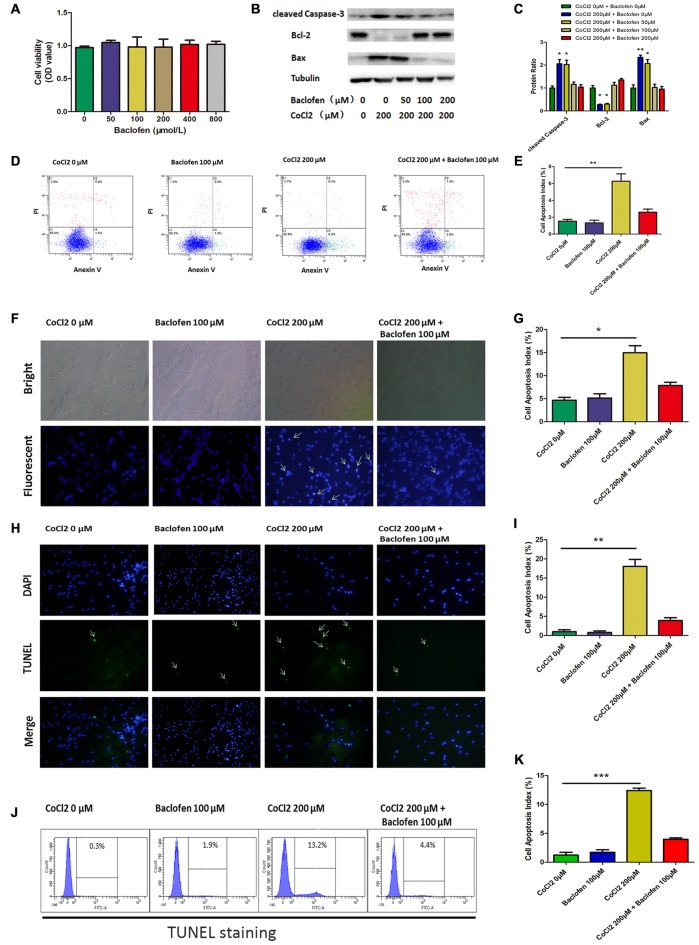
**Baclofen protected RGCs against CoCl_2_-induced apoptosis without interfering cell viability of RGCs. (A)** Cell viability detected by CCK-8 assay in RGCs treated with indicated concentrations of baclofen for 24 h. Data are means ± SD of three independent experiments. *p < 0.05, **p < 0.01 vs. basal level. **(B,C)** Expression of cleaved caspase-3, bax and bcl-2 detected by Western blotting in RGCs treated with indicated concentrations of CoCl_2_ and baclofen for 24 h. The detected protein bands are normalized to the signal of α-tubulin. The protein levels were expressed as the value relative to the control. The data represent the mean ± SD of three independent experiments. (*p < 0.05, **p < 0.01 compared to control). **(D,E)** Cell apoptosis detected by Annexin V and PI staining methods in RGCs treated with indicated concentrations of CoCl_2_ and baclofen for 24 h. C4 quadrant (Annexin V + /PI−) indicates the percentage of apoptotic cells. Values represent the mean ± SD of three independent experiments. **P < 0.01 vs. basal level. **(F,G)** RGCs stained with Hoechst nuclear stain after treatment with indicate concentrations of CoCl_2_ and baclofen for 24 h, and representative experiments are shown. Values represent the mean ± SD of three independent experiments. *P < 0.05 vs. basal level. White arrows indicate the apoptotic nuclei. **(H,I)** Terminal deoxynucleotidyl transferase-mediated dUTP nick end-labeling (TUNEL) staining was used to evaluate hypoxia-induced cell death. Cells were exposed to indicate concentrations of CoCl_2_ and baclofen for 24 h. Apoptotic nuclei were visualized by TUNEL. Representative experiments are shown (Final magnification, ×40). Values represent the mean ± SD of three independent experiments. **P < 0.01 vs. basal level. White arrows indicate the apoptotic nuclei. **(J,K)** TUNEL-positive cells detected by flow cytometry. Representative experiments are shown. Values represent the mean ± SD of three independent experiments. ***P < 0.001 vs. basal level.

First, to determine whether baclofen could affect the survival of RGCs, CCK-8 assays were performed, and the results showed that cell viability was not significantly altered after 24 h of exposure to baclofen at concentrations up to 800 μM, which indicates that, under certain conditions, baclofen has no significant influence on RGC viability (Figure 2A). Furthermore, we explored the effects of baclofen on cobalt-challenged RGCs. We found that baclofen decreased the levels of cleaved caspase-3 and bax and increased the expression of bcl-2 compared with those of hypoxic RGCs without baclofen and that the effect was dose-dependent (Figures 2B,C). At a concentration of 100 μM, the levels of apoptosis-related proteins were changed significantly. The levels of cell apoptosis detected by annexin V/PI double-stained flow cytometry also showed that baclofen significantly counteracted hypoxia-induced apoptosis in RGCs (*P* < 0.01, Figures 2D,E). To further confirm the protective effects of baclofen against hypoxia-induced cell death, we used Hoechst staining to detect apoptotic characteristics and TUNEL staining to detect DNA fragmentation and cell death in hypoxia RGCs with or without baclofen treatment. We treated RGCs with 100 μM baclofen and 200 μM CoCl_2_ for 24 h before performing Hoechst and TUNEL staining. Baclofen significantly decreased the percentage of apoptotic cells detected by both Hoechst and TUNEL staining (Hoechst: *P* < 0.05, Figures 2F,G; TUNEL: *P* < 0.01, Figures 2H,I; *P* < 0.001, Figures 2J,K). Taken together, these results suggest that baclofen protects RGCs from hypoxia-induced apoptosis without disturbing cell viability.

### Phosphorylation of Akt is Reduced and the PERK-eIF2α-ATF4 Pathway is Activated in Hypoxia-Treated RGCs, and Baclofen can Reverse the Change

**Figure 3:**
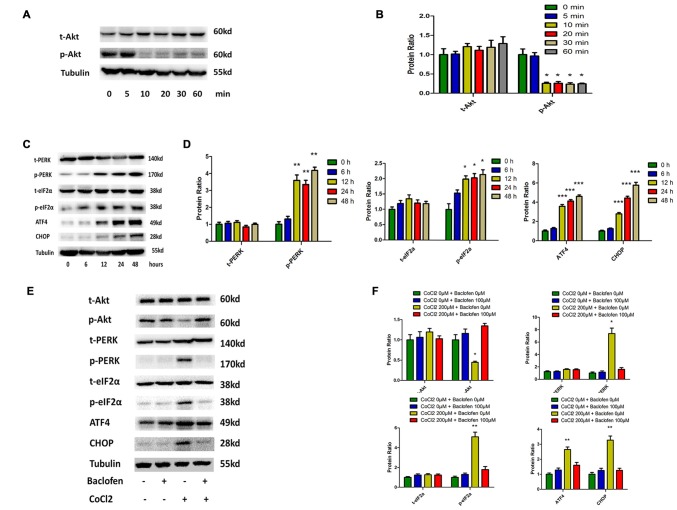
**Phosphorylation of Akt is reduced and protein kinase RNA (PKR)-like ER kinase (PERK)-eIF2α-ATF4 pathway is activated in hypoxia-treated RGCs, and baclofen can reverse the change. (A,B)** Time dependent expression of total Akt and phosphorylated Akt induced by 200 μM CoCl_2_. Representative experiments are presented. The detected protein bands are normalized to the signal of α-tubulin. Protein level in each group was expressed as the value relative to the control. The data represent the mean ± SD of three independent experiments. (*p < 0.05, compared to control). **(C,D)** Time dependent expression of indicated proteins induced by 200 μM CoCl_2_. **(E,F)** Expression of indicated proteins detected by Western blotting in RGCs treated with baclofen and CoCl_2_ for 24 h as presented. Protein expression is shown as percentage of control cells unexposed to chemotherapeutic drugs. The data represent the mean ± SD of three independent experiments (*p < 0.05, **p < 0.01, ***p < 0.001 compared to cells without CoCl_2_ or baclofen treatment).

The Akt pathway has been shown to be involved with various physiological and pathological process, including tumorigenesis and hypoxia (Di et al., 2015; Liu et al., 2015; Zhu et al., 2015). In RGCs, cobalt induced a significant decrease in the level of phosphorylated Akt without altering the total Akt expression level (Figures 3A,B). The rapid and transient decrease in Akt phosphorylation began at 10 min and lasted until 24 h after the application of the drug (Figures 3A,E). In previous studies, endoplasmic reticulum (ER) stress has been identified as a major mechanism of apoptosis in various types of cells (Chen et al., 2015a; Hiramatsu et al., 2015; López-Hernández et al., 2015). Our present study indicates that hypoxia induces ER stress by activating the PERK-eIF2α-ATF4 pathway. After 12 h of cobalt challenging, the levels of phosphorylated PERK, phosphorylated eIF2α, and ATF4 were significantly increased, while the levels of total PERK and total eIF2α were not significantly changed. In addition, the level of CCAAT/enhancer-binding protein homologous protein (CHOP) protein was found to be upregulated after 12 h of CoCl_2_ treatment (Figures 3C,D). The CHOP pathway is the best-characterized pathway that has been reported to be involved in ER stress-induced apoptosis (Li et al., 2014a; López-Hernández et al., 2015).

We then explored the beneficial effects of baclofen on hypoxia-mediated stress in RGCs. We pretreated RGCs with baclofen and with or without CoCl_2_ for 24 h and then determined the levels of the relevant proteins using western blot analysis. As described above, baclofen had no negative effect on RGC survival, and western blotting indicated that baclofen did not change the Akt, PERK-pathway or CHOP protein levels compared with those of the control group. In the hypoxia treatment group, the administration of baclofen significantly increased the phosphorylated Akt level and decreased the phosphorylated PERK, phosphorylated eIF2α, ATF4 and CHOP levels following hypoxic injury (Figures 3E,F).

### Baclofen Mediated RGC Apoptosis via the GABA_B_ Receptor

**Figure 4:**
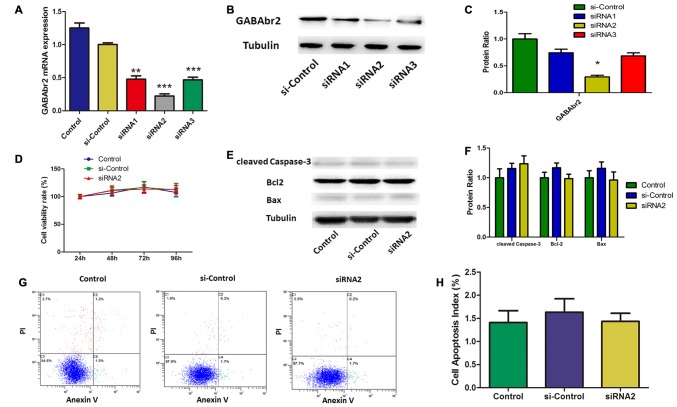
**Small interfering RNA (siRNA) silenced GABA_B_2 expression without interfering RGCs survival. (A–C)** GABA_B_2 mRNA expression **(A)** and GABA_B_2 protein level **(B,C)**. Up on transfected with siRNAs against GABA_B_2 and control siRNA. **p < 0.01, ***p < 0.001 vs. basal level. **(D)** Time course of cell viability detected by CCK-8 assay in RGCs transfected with indicated siRNAs. Cell viability was quantified based on three independent experiments (mean ± SD). **(E,F)** Expression of cleaved caspase-3, bax and bcl-2 detected by Western blotting in RGCs transfected with indicated siRNAs. The detected protein bands are normalized to the signal of α-tubulin. The level of protein in each group was expressed as the value relative to the control. The data represent the mean ± SD of three independent experiments. (*p < 0.05, vs. control). **(G,H)** Cell apoptosis detected by Annexin V and PI staining methods in RGCs transfected with indicated siRNAs. C4 quadrant (Annexin V + /PI−) indicates the percentage of apoptotic cells. Representative experiments are shown. Values represent the mean ± SD of three independent experiments.

Baclofen is an agonist of the GABA_B_ receptor. To understand the role of the GABA_B_ receptor in the baclofen-mediated protection from apoptosis for hypoxic RGCs, GABA_B_2 was knocked down by short interfering (si) RNA. Both qRT-PCR and western blot assays showed that siRNA2 knockdown of GABA_B_2, for both RNA and protein levels, was more effective than that of siRNA1 and siRNA3 (Figures 4A–C). To determine the effect of GABA_B_2 knockdown on cell viability and apoptosis, CCK8 assays, western blotting and annexin V/PI double-stained flow cytometry were performed. Cell viability was not significantly changed by GABA_B_2 silencing (Figure 4D). Flow cytometry (Figures 4E,F) and western blot analysis (Figures 4G,H) showed that GABA_B_2 depletion had no effect on RGC apoptosis.

**Figure 5:**
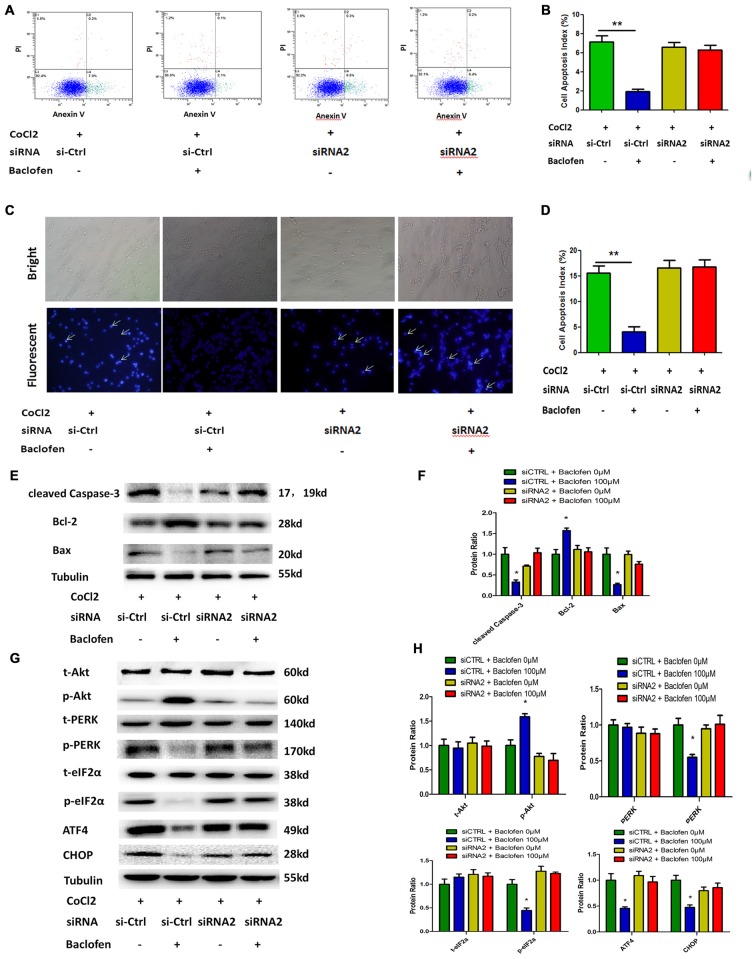
**Baclofen mediated RGCs apoptosis via GABA_B_ receptor. (A,B)** RGCs were transfected with GABA_B_2 siRNA2 or control siRNA before treatment with CoCl_2_ and baclofen for 24 h as indicated. Cell apoptosis detected by Annexin V and PI staining methods. C2, C4 quadrant (Annexin V + /PI−) indicate the percentage of necrotic or apoptotic cells, respectively. Values represent the mean ± SD of three independent experiments. **P < 0.01 vs. basal level. **(C,D)** RGCs stained with Hoechst nuclear stain after treatment as described above. White arrows indicate the apoptotic nuclei. Values represent the mean ± SD of three independent experiments. *P < 0.05 vs. basal level. **(E–H)** RGCs were transfected with GABA_B_2 siRNA2 or control siRNA before treatment with CoCl_2_ and baclofen. Expression of apoptosis related proteins **(E,F)** and pathway related proteins **(G,H)** detected by Western blotting treated with baclofen and CoCl_2_ for 24 h as presented. α-tubulin served as a loading control. The level of protein in each group was expressed as the value relative to the control. The data represent the mean ± SD of three independent experiments. (*p < 0.05, vs. control).

As observed in the hypoxia-treated RGCs, flow cytometry results indicated that GABA_B_2 depletion abolished the baclofen-induced decrease in hypoxia-induced RGC apoptosis (Figures 5A,B). Hoechst staining revealed similar results and indicated that the knockdown of GABA_B_2 decreased baclofen’s protective effect on hypoxic RGCs compared the effect observed in the siRNA-control group (Figures 5C,D). The expression levels of cleaved caspase-3, bax and bcl-2 in the GABA_B_2-knockdown hypoxic RGCs treated with baclofen were similar to those without baclofen treatment. In the siRNA-control groups, baclofen significantly reduced the levels of cleaved caspase-3 and bax and increased the level of bcl-2 in hypoxic RGCs (Figures 5E,F). We next explored the relationship between the GABA_B_ receptor and Akt, the PERK-eIF2α-ATF4 pathway and CHOP. In GABA_B_2-depleted RGCs, baclofen did not significantly change the levels of Akt, PERK-pathway and CHOP proteins under hypoxic conditions compared with the levels in the baclofen-free group. However, in the siRNA-control RGCs, the administration of baclofen significantly increased the phosphorylation of the Akt protein and decreased the levels of phosphorylated PERK, phosphorylated eIF2α, ATF4 and CHOP after hypoxic injury, compared with the levels of the baclofen-free group (Figures 5G,H). These data indicate that the GABA_B_ receptor is required for the baclofen-induced protective effect on hypoxic RGCs.

### Inhibition of Akt Phosphorylation Abolished the Protection by Baclofen Against Hypoxia-Induced Apoptosis in RGCs

**Figure 6:**
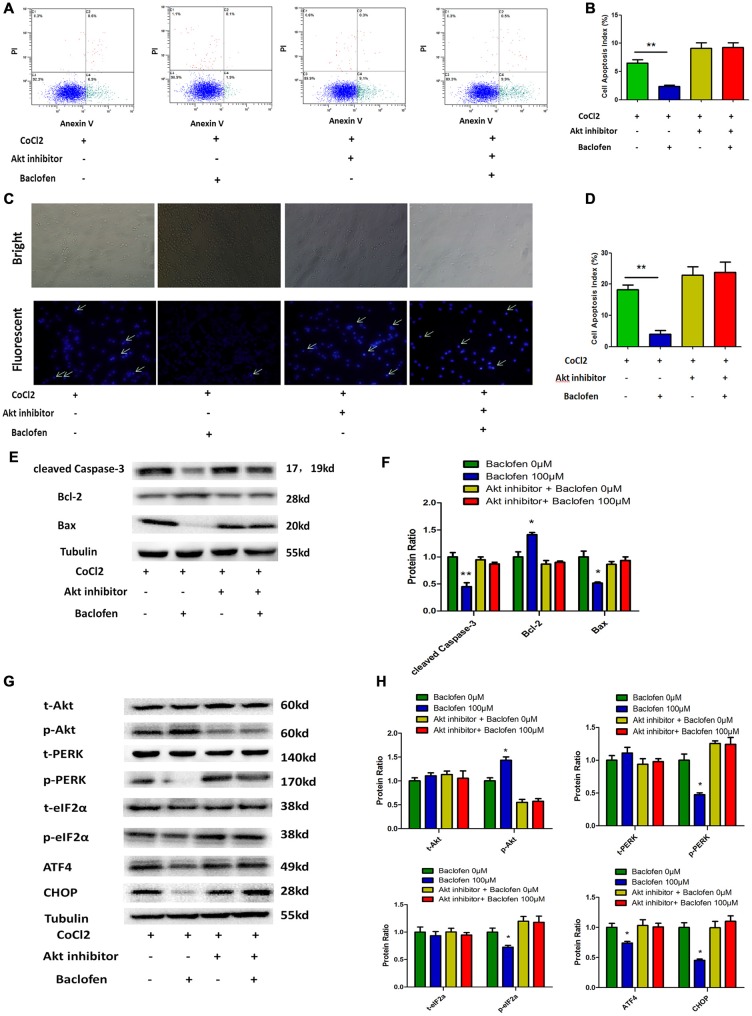
**Akt phosphorylation is needed for baclofen mediating RGCs apoptosis. (A,B)** Cell apoptosis detected by Annexin V and PI staining methods in RGCs treated with CoCl_2_, baclofen and Akt inhibitor for 24 h as indicated. C4 quadrant (Annexin V + /PI−) indicates the percentage of apoptotic cells. Values represent the mean ± SD of three independent experiments. **P < 0.01 vs. basal level. **(C,D)** RGCs stained with Hoechst nuclear stain after treatment as described above. White arrows indicate the apoptotic nuclei. Values represent the mean ± SD of three independent experiments. **P < 0.01 vs. basal level. **(E–H)** Expression of apoptosis related proteins **(E,F)** and pathway related proteins **(G,H)** detected by Western blotting in RGCs treated with baclofen, CoCl_2_ and Akt inhibitor for 24 h as presented. α-tubulin was included as a loading control. Protein levels were expressed as the value relative to the control in each group. The data represent the mean ± SD of three independent experiments. (*p < 0.05, vs. control).

As mentioned above, the Akt pathway was shown to be activated during the protection by baclofen against hypoxia-induced apoptosis in RGCs. To understand the role of the activation of the Akt pathway in this protective process, an inhibitor of Akt phosphorylation (MK-2206 2HCl) was applied. According to the manufacture, MK-2206 2HCl blocks Akt phosphorylation without changing the total Akt level. Annexin V/PI double-stained flow cytometry results indicated that baclofen did not significantly reduce the percentage of apoptotic cells in the RGCs treated with MK-2206 2HCl compared with that in the CoCl_2_-treated group. Meanwhile, in the RGCs without MK-2206 2HCl treatment, baclofen significantly reduced the percentage of apoptotic cells compared with that in the CoCl_2_-treated group (*P* < 0.01; Figures 6A,B). Hoechst assays showed that the inhibition of Akt phosphorylation failed to reduce the percentage of apoptotic cells compared with that in the CoCl_2_-treated group (Figures 6C,D). Western blotting demonstrated that inhibiting Akt phosphorylation in hypoxic RGCs did not significantly change the levels of cleaved caspase-3, bax and bcl-2 regardless of baclofen treatment (Figures 6E,F). Moreover, western blot assays indicated that the inhibition of Akt phosphorylation increased the basal levels of PERK phosphorylation in hypoxic RGCs, as well as those of phosphorylated eIF2α, ATF4 and CHOP, which were not further decreased after baclofen treatment (Figures 6G,H). Together, these results indicate that Akt phosphorylation is necessary for baclofen’s protective effect against hypoxia-induced apoptosis and the activation of ER stress in RGCs.

## Discussion

Hypoxia, *in vitro* and *in vivo*, triggers mixed cell death due to both necrosis and apoptosis. Previously, studies have indicated that baclofen has pharmacological effects against cell apoptosis in various types of cells (Naseer et al., 2013; Tian et al., 2013; Liu et al., 2015). However, no data have yet been reported in an *in vitro* experiment related to RGCs. Thus, we investigated the use of an *in vitro* model of purified rat RGC cultures to examine whether baclofen has neuroprotective effects against RGC apoptosis induced by hypoxic stress.

It has been reported that ganglion cells in diabetic retinas express several proapoptosis molecules, suggesting that these cells are among the most vulnerable neurons (Abu-El-Asrar et al., 2004). Following study revealed that in superoxide dismutase 1 (SOD1)-deficient mice, RGC degeneration precedes the degeneration of other layers in retina, indicating the greater vulnerability of RGCs to oxidative stress compared with other neurons (Yuki et al., 2011).

While it is difficult to control oxygen levels precisely to simulate hypoxic condition in cell culture, various *in vitro* models of neuronal hypoxia have been provided using divalent cations such as cobalt (Montel et al., 2007; Hang et al., 2009; Lopez-Sánchez et al., 2014; Yang et al., 2016). Cobalt have been applicable to mimic hypoxic conditions in cultured cells because they activate hypoxic signals by stabilizing the expression of hypoxia-inducible factor-1 alpha (HIF-1α), an O_2_-regulated transcriptional activator. Studied have verified that incubation of cells with CoCl_2_ mimics the hypoxia situation confirmed by the observed increase of VEGF protein expression and secretion vs. normoxia (Carbajo-Pescador et al., 2013). Increased HIF-1α leval in cells cultured in hypoxia-mimetic agent treated cells are similar to those observed in a hypoxia chamber (1% O_2_; Liu et al., 2014). Thus in this study, CoCl_2_ was added at a final concentration of 200 mM to mimic hypoxia. We demonstrated that mimicking hypoxia *in vitro* by using CoCl_2_ is capable of inducing apoptosis in primary RGC cell culture in a dose dependent way.

Based on the current results, we showed that baclofen protects RGCs against apoptosis via the GABA_B_ receptor in hypoxic culture conditions. The GABA_B_ receptor is an allosteric complex made of two subunits, GABA_B_1 and GABA_B_2. GABA_B_2 plays a major role in coupling to G proteins, whereas GABA_B_1 binds GABA. GABA_B_2’s association with GABA_B_1 plays a crucial role in the agonist-induced GABA_B_ receptor activation (Liu et al., 2004; Pin et al., 2004). Thus, the depletion of GABA_B_2 abolished the baclofen-induced GABA_B_ receptor activation and prevented the GABA_B_ receptor from binding with the G protein, which prevented the subsequent signaling pathway cascade. In the present study, we demonstrated that the siRNA-mediated knockdown of GABA_B_2 inhibits the protective effect of baclofen on RGCs in cobalt-challenged condition, which confirmed the specific involvement of the GABA_B_ receptor in the protective effect of baclofen.

Moreover, our present study demonstrates that baclofen effectively prevented hypoxia-induced apoptosis by downregulating the expression of cleaved caspase-3 and bax and increasing the expression of bcl-2 in RGCs. The hypoxia-induced caspase-3 activation, bax upregulation and bcl-2 downregulation in various cells are reversed by the activation of the PI3K/Akt signaling pathway, which leads to the acquisition of antiapoptotic properties (Mounir et al., 2011; Chen et al., 2015b; Zhang et al., 2015). The expression of antiapoptotic and proapoptotic proteins, especially caspase-3, bax and bcl-2, determines the susceptibility to apoptosis (Wei et al., 2001; Sadidi et al., 2009). We therefore investigated whether these pathways were involved in the antiapoptotic effects of baclofen in RGCs. The caspase-3 and bcl-2/bax apoptotic signaling pathways mediate the protective effects of baclofen in neurons (Tyurenkov et al., 2012; Li et al., 2014b), lung tissue (Jin et al., 2015) and pancreatic β-cells (Tian et al., 2013). Our result revealed that baclofen reduced caspase-3 activity and increased the bcl-2/bax level, suggesting that baclofen inhibits RGC apoptosis through the regulation of the activity of caspase-3 and of bcl-2/bax expression, which is consistent with the results of previous studies. Moreover, we found that Akt and the PERK and CHOP pathways were involved in the protective effects of baclofen.

In this study, we demonstrated that, in hypoxic RGCs, the activity of Akt was much higher in the baclofen-treated group than that in the hypoxic control group. Numerous studies have shown that the phosphatidylinositol 3-kinase–Akt (PI3K/AKT) signaling pathway plays a major role in cell apoptosis (Tu et al., 2010; Shilpa et al., 2013; Purwana et al., 2014; Di et al., 2015; Liu et al., 2015). The specific involvement of the GABA_B_ receptor in Akt activation has been investigated in several types of cells. It has been reported that the PI3K/AKT signaling pathway is altered when retinal cells are exposed to oxidative stress, which can subsequently alter downstream signaling cascades (Wang et al., 2008). In cerebellar granule neurons, the GABA_B_ receptor agonist baclofen protect neurons from apoptosis through the regulation of bcl-2/bax or caspase-3 via the PI3K/Akt pathway (Tu et al., 2010; He et al., 2014; Liu et al., 2015). To understand whether the increase in Akt phosphorylation is necessary for the protective effects of baclofen on RGCs, an inhibitor of Akt phosphorylation was applied. Inhibition of Akt phosphorylation abolished the protection by baclofen against hypoxia-induced apoptosis in RGCs, and the levels of cleaved caspase-3, bax and bcl-2 did not change significantly when Akt phosphorylation was blocked regardless of baclofen treatment. These data demonstrate that Akt activation is one of the essential processes involved in the neuroprotective effect of baclofen on hypoxic RGCs.

Apoptosis is tightly controlled by a variety of signaling pathways that either promote or inhibit the apoptotic cascades. Recent evidence suggests that disturbed protein homeostasis and ER stress contribute to apoptosis in retinal cells (Jing et al., 2012). Several lines of evidence point to a strong relationship between hypoxia and the accumulation of misfolded proteins in the ER (Hiramatsu et al., 2015) leading to the unfolded protein response (UPR), which is toxic to cells. Moreover, ER stress activates a large number of genes involved in the control of cell fate, including antiapoptotic and proapoptotic molecules such as bax and bcl-2 (Mounir et al., 2011). Therefore, we clarified the role of ER stress in RGC apoptosis, as well as the relationship between baclofen’s protective effect and ER stress.

Three ER-related proteins, PERK (Harding et al., 1999), activating transcription factor 6 (ATF6; Ron and Walter, 2007) and inositol-requiring enzyme-1 (IRE1; Tirasophon et al., 1998; Calfon et al., 2002), are involved in the initial signaling to the cell through the UPR. Initially, ER stress leads to adaptations to the changing environment and the restoration of normal ER function. However, during prolonged or overwhelming ER stress, the UPR fails to restore normal ER function, and apoptotic cascades can be activated (Sano and Reed, 2013; Williams et al., 2014).

Previous studies demonstrated that increased ER stress in apoptotic RGC degeneration is accompanied by increased ER stress-related proteins, such as Bip, PERK and CHOP (Shimazawa et al., 2007; Doh et al., 2010). Our present research indicates that hypoxia-induced ER stress was initiated in RGCs via the activation of the PERK pathway, which could be reduced by baclofen treatment, while ATF6 and IRE1 levels were not significantly changed (data not shown). Furthermore, both the depletion of the GABA_B_ receptor and the inhibition of Akt activation increased the basal levels of the PERK-pathway proteins (PERK/eIF2α/ATF4) in cobalt-treated RGCs, which were not decreased following baclofen treatment. Together, these data indicate that baclofen protects RGCs from hypoxia-induced apoptosis by downregulating the PERK pathway, which is regulated by the GABA_B_ receptor and the Akt pathway.

CHOP is a major stress-inducible proapoptotic gene involved in ER stress-induced apoptosis (Wang and Ron, 1996). All three branches of the UPR regulate the activation of CHOP (Li et al., 2014a); however, ATF4 is considered to be the major inducer of CHOP expression (Jing et al., 2012; Li et al., 2014a). Recent studies show that both the antiapoptotic gene bcl-2 and the proapoptotic protein bax (apoptosis-associated proteins that localize on the mitochondrial membrane) are regulated by CHOP during ER stress (McCullough et al., 2001; Puthalakath et al., 2007). This provides evidence that the proapoptotic functions of CHOP are associated with mitochondria-dependent mechanisms of cell death. Our data support this premise, which was substantiated by the observations of increased caspase-3 activity in hypoxia-treated RGCs. CHOP was down regulated by baclofen in hypoxic RGCs, however, this down regulation was abolished by the depletion of the GABA_B_ receptor or the inhibition of Akt activation.

In conclusion, this study demonstrates the beneficial effects of baclofen, a GABA_B_ receptor agonist, in the treatment of hypoxia-induced apoptosis in RGCs. Baclofen exerted its effects in protecting hypoxic RGCs from apoptosis by activating the GABA_B_ receptor, upregulating Akt phosphorylation and downregulating the PERK pathway during ER stress, as well as by downregulating CHOP, which finally downregulated the levels of the proapoptotic proteins cleaved caspase-3 and bax and upregulated the antiapoptotic protein bcl-2. These results identify baclofen as an effective regulator of Akt and PERK signaling in RGCs, and offers insight on the role of baclofen in regulating the neuron survival in cellular pathology associated with hypoxic ischemic disorders of retina. Baclofen could be a potentially useful therapeutic drug for the treatment of retinal hypoxia diseases.

## Author Contributions

QW conceived and directed the study. PF, TL and QW contributed to the project design. PF, JH, TL and FG performed experiments. PF, TL and FG performed bioinformatics data analysis. JH contributed samples, data and comments on the manuscript. PF, TL and FG analyzed and interpreted data. PF drafted the manuscript.

## Conflict of Interest Statement

The authors declare that the research was conducted in the absence of any commercial or financial relationships that could be construed as a potential conflict of interest.
